# A mini-review of quality of life as an outcome in prostate cancer trials: patient-centered approaches are needed to propose appropriate treatments on behalf of patients

**DOI:** 10.1186/s12955-018-0870-6

**Published:** 2018-03-05

**Authors:** Yohann Foucher, Marine Lorent, Philippe Tessier, Stéphane Supiot, Véronique Sébille, Etienne Dantan

**Affiliations:** 1 SPHERE (MethodS for Patients-centered outcomes and HEalth Research), INSERM UMR 1246, Nantes University, IRS2 - 22 boulevard Bénoni Goullin, 44200 Nantes, France; 2 CHU Nantes University Hospital, Nantes, France; 3 ICO - Institut de Cancérologie de l’Ouest - Centre René Gauducheau, Boulevard Jacques Monod, 44805 Saint-Herblain, France

**Keywords:** Prostate cancer, Review of clinical trials, Patient-centered outcomes, Quality of life

## Abstract

**Background:**

Patients with prostate cancer (PC) may be ready to make trade-offs between their quantity and their quality of life. For instance, elderly patients may prefer the absence of treatment if it is associated with a low-risk of disease progression, compared to treatments aiming at preventing disease progression but with a substantial deterioration of their Health-Related Quality of Life (HRQoL). Therefore, it seems relevant to compare the treatments by considering both survival and HRQoL. In this mini-review, the aim was to question whether the potential trade-offs between survival and HRQoL are considered in high impact factor journals.

**Methods:**

The study was conducted from the PubMed database for recent papers published between May 01, 2013, and May 01, 2015. We also restricted our search to nine medical journals with 2013 impact factor > 15.

**Results:**

Among the 30 selected studies, only six collected individual HRQoL as a secondary endpoint by using the Functional Assessment of Cancer Therapy-Prostate (FACT-P) questionnaire. In four studies, the time to HRQoL change was analyzed, but its definitions varied. In two studies, the mean changes in HRQoL between the baseline and the 12- or 16-week follow-up were analyzed. None of the six studies reported in a single endpoint both the quantity and the quality of life.

**Conclusions:**

Our mini-review, which only focused on recent publications in journals with high-impact, suggests moving PC clinical research towards patient-centered outcomes-based studies. This may help physicians to propose the most appropriate treatment on behalf of patients. We recommend the use of indicators such as Quality-Adjusted Life-Years (QALYs) as principal endpoint in future clinical trials.

**Electronic supplementary material:**

The online version of this article (10.1186/s12955-018-0870-6) contains supplementary material, which is available to authorized users.

## Background

In Europe, Prostate Cancer (PC) is the second most frequent cancer in men with an incidence of 9·55 per 1000 person-years when an invitation to screening is performed and 6·23 per 1000 person-years otherwise [1]. Early diagnosis improved by PSA testing has recently allowed a better estimation of its incidence [2]. Over the last decades, many progresses have been done in the treatment of patients with PC, partially explained by the improvement of the prediction of the disease progression based on scoring systems [3, 4]. The objectives of assessing PC patients’ risk level of future adverse health events is i) to avoid over-treatment of patients at low-risk of recurrence or death related to PC, and ii) to avoid under-treatment of high-risk patients.

Although guidelines are available for such stratified medical decision making [5, 6], some questions remain unresolved. One of the main issues to address concerns the trade-offs between the benefits and the costs of possible treatment options in terms of both survival and health-related quality of life (HRQoL). Several studies have shown that PC patients are ready to make trade-offs between their quantity and their quality of life [7–10], especially when providing balanced information of different treatment options [11]. For instance, elderly patients may never experience disease progression to metastatic stage during their remaining lifetime [12], while treatments aiming at preventing disease progression can substantially deteriorate their HRQoL [13]. Younger men may also prefer interventions that preserve their HRQoL, but at the potential cost of reducing the disease progression-free survival. In a patient-centered medical decision making perspective, the treatments should therefore be compared against each other by weighting their benefits and costs in terms of both survival and HRQoL.

In this context, we proposed a mini-review. This type of study provides a focused review of the literature, the main objective being to raise questions or to suggest new hypotheses for research. We aimed to question whether the trade-offs between survival and HRQoL are considered in high-impact factor journals and to suggest recommendations for future studies based on patient-centered endpoints.

## Methods

### Literature search strategy

A literature search was conducted from the PubMed database for recent papers published between May 01, 2013, and May 01, 2015. In order to obtain a picture of the main trends in the medical literature, we focused on nine prominent journals in oncology or general medicine (impact factor ≥ 15 in 2013). We indicated « prostatic neoplasms » as Medical Subject Headings (MeSH) terms and « randomized controlled trial » as publication type. The research equation used in PubMed is presented in Additional file 1. The PRISMA-P (Preferred Reporting Items for Systematic review and Meta-Analysis Protocols) checklist is also provided in the Additional file 2.

### Data extraction

All papers resulting from this search were independently double-blinded reviewed (Y Foucher, M Lorent, or E Dantan). The first task was to exclude papers associated with non-randomized controlled trials, non-original works, without patients’ follow-up, or non-comparative analyses. The second task was to collect the following characteristics from the selected papers: the study design, the patients’ inclusion criteria, the patients’ maximum follow-up duration, the compared treatments, the sample size in each arm, the endpoints, the statistical methods used, the reference to the results of an additional paper and the financial support. If any disagreements between reviewers occurred, they were solved by discussions. We used Zotero to manage the records.

## Results

### Retained studies

**Fig. 1:**
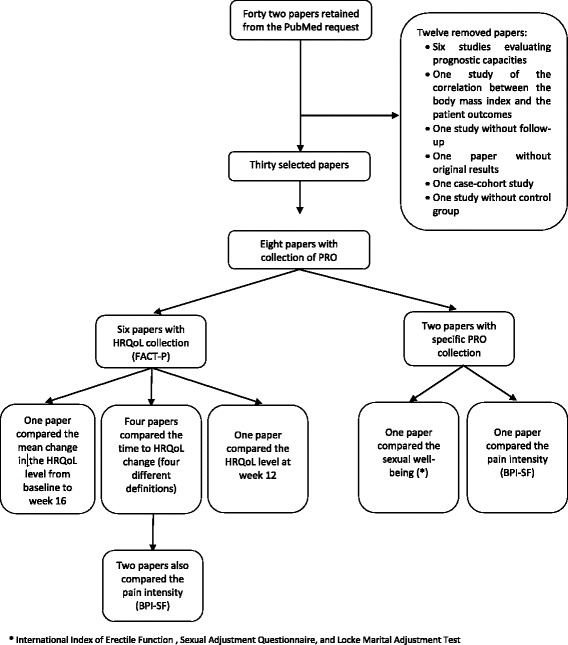
Flow diagram of the literature search strategy and the used patient reported outcomes

The PubMed request allowed identifying 42 papers (Fig. 1). Because we only considered randomized clinical trials comparing at least two interventions, 12 publications were excluded: six re-analyses of clinical trials evaluating the prognostic capacities of markers or models [14–19]; one study related to body mass index (no comparison of treatments) [20]; one study without patients’ follow-up [21]; one paper without original results [22]; one case-cohort study [23]; one study without control group [24]; and one diagnostic study [25]. Finally, 30 papers [1, 26–54] were retained and are described in Table 1. As detailed in the last column entitled “other results”, two papers referred to the trial NCT00887198 [27, 47] and three papers referred to the trial NCT00699751 [37, 42, 48].

**Table 1:** Descriptive of the 30 studies aiming to compare at least two interventions in a population of patients with or susceptible to have PC

Study	Design	Inclusion criteria	Treatments	Primary endpoints	Secondary endpoints	HRQoL analysis	Other results
[26]	multicentric, double-blind, phase 3, 2008–2011, follow-up of 48 months	adult, chemotherapy-naive, metastatic, castration-resistant PC, adequate organ function	dasatinib (*n* = 762) vs placebo (*n* = 760)	overall survival	proportion who achieved an objective response, time to first skeletal-related event, proportion with reduction > 35% in urinary N-telopeptide from baseline, progression-free survival, time to PSA progression, and proportion with reduction > 30% in pain intensity from baseline BPI-SF	stratified Cochran-Mantel-Haenszel	no
[27]	multicentric, double-blind, phase 3, 2009–2010, follow-up of 30 months	progressive, metastatic castration-resistant PC, BPI-SF score of zero to three, not previous chemotherapy	abiraterone (*n* = 546) vs placebo (*n* = 542)	time to pain progression (BPI-SF): increase ≥30% from baseline of the mean pain intensity, or increase ≥30% from baseline of the worst pain intensity, or increase ≥50% from baseline of the interference pain intensity	time to HRQoL deterioration (FACT-P) from baseline: decrease of the total score > 10%, or decrease of the general function > 9%, or decrease of the trial outcome index > 9%, or decrease of the prostate-specific-subscale > 3%, or decrease of the general function subscales > 3%	Kaplan-Meier	[47]
[28]	multicentric, double-blind, phase 3, 2010–2012, follow-up of 18 months	metastases and PSA progression, radiographic progression, or both in bone or soft tissue, despite receiving luteinizing hormone-releasing hormone analogue therapy or undergoing orchiectomy, serum testosterone level < 1·73 nmol/L, ECOG grade 0–1, BPI-SF score 0–3	enzalutamide (*n* = 872) vs placebo (*n* = 845)	radiographic progression-free survival and overall survival	time to initiation of cytotoxic chemotherapy, time to first skeletal-related event, best overall soft-tissue response, time to PSA decline > 50% or more from baseline, time to decline in QoL (FACT-P)	Kaplan-Meier	no
[29]	multicentric, open-label, phase 3, 1989–1999, follow-up of 18 years	younger than 75 years of age, life expectancy > 10 years, no other known cancers, localized tumor (T0d, T1, or T2, 1978 criteria of the International Union against Cancer)	radical-prostatectomy (*n* = 347) vs watchful-waiting (*n* = 348)	death from any cause, death from PC, and the risk of metastases	initiation of androgen deprivation therapy	no	no
[30]	multicentric, double-blind, phase 3, 1997–2010, follow-up of 24 months	radical prostatectomy for clinically localized (T1c or T2) PC less than 4 months before randomization, post-surgery PSA < 0.07 ng/mL confirmed by the assay used in this study, and fulfilled one or more following criteria: preoperative PSA > 20.0 ng/mL, final Gleason score ≥ 8, positive surgical margins, extracapsular extension, seminal vesicle invasion, or micro metastases in any removed pelvic lymph nodes	soy protein supplement (*n* = 86) vs placebo (*n* = 90)	2-year rate of biochemical cancer recurrence and time to recurrence for those in whom cancer recurred	adverse events (gastrointestinal, urinary tract, initiation of cholesterol or hypertension treatments, musculoskeletal pain)	no	no
[31]	multicentric, open-label, phase 3, 1998–2001, follow-up of 10 years	adult, histologically confirmed (T1b–T3a, N0, M0), PSA < 50 ng/mL, no contraindications for radical radiotherapy	escalated-dose (*n* = 422) vs control-doses (*n* = 421) radiotherapy	Biochemical progression-free survival and overall survival	biochemical failure, initiation of androgen deprivation therapy, progression-free survival, metastases-free survival, time to PC or non-PC death	no	no
[32]	multicentric, open-label, phase 3, 2003–2007, follow-up of 7 years	locally advanced PC (either T2a N0 M0, PSA ≥10 μg/L, Gleason score of ≥7) or T2b–4 N0 M0 tumors regardless of PSA and Gleason score	androgen suppression + leuprorelin (*n* = 268) or zoledronic acid (n = 268) or both (*n* = 267) vs standard (n = 268)	PC-specific mortality	PSA progression, local progression, distant progression, bone progression, time to secondary therapeutic intervention, all-cause mortality	no	no
[33]	multicentric, double-blind, phase 3, 2008–2011, follow-up of 38 months	adult, histologically or cytologically confirmed PC, surgically castrated or continuously medically castrated, serum testosterone levels ≤ 2·4 nmol/L	oral zibotentan (524) vs placebo (528)	overall survival	time to pain and PSA progression, progression-free survival, safety, time to deterioration of symptoms (FACT-P Symptom Index-8), time to deterioration of HRQoL (FACT-P total scores)	Log-rank	no
[34]	multicentric, open-label, phase 2, 2011–2013, 108 weeks	progressive metastatic castration-resistant PC, castrate concentrations of testosterone, ECOG grade 0–1	1400 (*n* = 35) or 400 (*n* = 37) mg daily doses of ODM-201 vs standard (*n* = 38)	PSA response at week 12, defined as a decrease of 50% or more in serum PSA from baseline	objective disease response (RECIST for soft tissue and PCWG2 criteria for bone), time to PSA progression analyzed (PCWG2 criteria), and time to radiographic disease progression (RECIST and PCWG2 criteria)	no	no
[35]	multicentric, double-blind, phase 3, 2009–2010, follow-up of 24 months	histologically or cytologically confirmed PC, undergone orchiectomy or received gonadotropin-releasing hormone agonist therapy, castrate testosterone levels, ECOG grade 0–2, previously received docetaxel, progressive disease (PCWG2 criteria)	enzalutamide (*n* = 800) vs placebo (*n* = 399)	overall survival	time to first skeletal-related event, change in pain severity and interference from baseline to week 13, pain palliation at week 13, pain progression at week 13, time to pain progression, overall improvement in HRQoL, improvements in individual HRQoL domains, time to HRQoL deterioration (FACT-P)	Kaplan-Meier, Log-Rank, stratified Cochran-Mantel-Haenszel	no
[54]	multicentric, double-blind, phase 3, follow-up of 30 months	adult, histologically or cytologically confirmed PC, radiographically documented metastatic disease with evidence of disease progression after receiving docetaxel	orteronel (*n* = 734) vs placebo (*n* = 365)	overall survival	radiographic progression-free survival, PSA decrease > 50% at 12 weeks, pain response at 12 weeks, response by RECIST 1·1, time to PSA progression, duration of pain response, time to pain progression, safety	no	no
[36]	simulation-based study, follow-up of 11 years	between the ages of 55 and 75 years	screening vs non-screening	incremental cost-effectiveness ratio	life-years gained, costs, gained in quality-adjusted life-years	no	no
[37]	multicentric, double-blind, phase 3, 2008–2011, follow-up of 36 months	histologically confirmed, progressive castration-resistant PC, two or more bone metastases detected on skeletal scintigraphy and no known visceral metastases, baseline PSA level ≥ 5 ng/mL, evidence of progressively increasing PSA values, ECOG grade 0–2, life expectancy ≥ 6 months, adequate hematologic, renal, and liver function	radium-223 (n = 614) vs placebo (n = 307)	overall survival	time to first symptomatic skeletal event, time to increase in PSA concentration, time to increase in total alkaline phosphatase concentration, confirmed reduction of at least 30% in total alkaline phosphataseponse, normalization of total alkaline phosphatase concentration	no	[42, 48]
[38]	multicentric, open-label, phase 3, 1996–1999, follow-up of 12 years	born between 1929 and 1944 (aged 55, 59, 63, or 67 years at entry), identified from the Finnish population Registry, no previous PC diagnosis	screening (*n* = 31,866) vs non-screening (*n* = 48,278)	overall mortality and prostate-specific mortality	no	no	no
[39]	multicentric, double-blind, phase 3, 2009–2012, follow-up of 36 months	at least one bone metastasis from castration-resistant PC, progression after docetaxel treatment	ipilimumab (n = 399) vs placebo (*n* = 400)	overall survival	progression-free survival, pain response, safety profile	no	no
[40]	multicentric, double-blind, phase 3, 2008–2010, follow-up of 36 months	histologically or cytologically confirmed PC, metastatic and castration-resistant (refractory to androgen ablation), surgical or ongoing chemical castration, baseline testosterone level < 50 ng/dL	sunitinib (*n* = 584) vs placebo (*n* = 289)	overall survival	progression-free survival, objective response rate, safety	no	no
[41]	open-label, pilot study, follow-up of 3 months	localized PC (T1c-T3,N0/NX), Gleason score > 6, risk of nodal involvement > 10%, no prior therapy for PC, no history of thrombosis, no unstable angina, no heart failure, serum testosterone > 280 ng/dL, normal blood counts, creatinine, and transaminases	goserelin (*n* = 12) or bicalutamide (*n* = 10) + dutasteride vs bicalutamide, dutasteride, and ketoconazole (*n* = 13)	Prostate tissue androgen levels	serum androgen levels, prostate androgen receptor program activity, PSA nadir, pathologic outcomes	no	no
[42]	multicentric, double-blind, phase 3, 2008–2011, follow-up of 38 months	histologically confirmed, progressive castration-resistant PC, two or more bone metastases detected on skeletal scintigraphy and no known visceral metastases, baseline PSA level ≥ 5 ng/mL, evidence of progressively increasing PSA values, ECOG grade 0–2, life expectancy ≥ 6 months, adequate hematologic, renal, and liver function	radium-223 (*n* = 614) vs placebo (*n* = 307)	overall survival	time to the first symptomatic skeletal event, clinically evaluated symptomatic skeletal events, total alkaline phosphatase and PSA concentrations, adverse events, mean change in the FACT-P total score at week 16	Student	[37, 48]
[43]	multicentric, double-blind, phase 3, 2009–2012, follow-up of 52 weeks	adult, clinical stage II (T1b-T2bN0M0) PC, with i) Gleason score < seven and the serum PSA level < 20 ng/mL, or ii) Gleason score ≥ seven and PSA < 15 ng/mL, ability to achieve erection at least half the time (International Index of Erectile Function question one response 3–5), Zubrod Performance Status score < 2, serum testosterone within normal limits, and no prior bilateral orchiectomy, chemotherapy, external radiotherapy, brachytherapy, surgical, or other ablative therapy for PC	tadalafil (*n* = 121) vs placebo (*n* = 31)	maintaining spontaneous erections between weeks 28 and 30 after the start of radiotherapy	overall sexual function, sexual satisfaction, marital adjustment and their partners’ sexual satisfaction, adverse events (NCI Common Terminology Criteria for Adverse Events, version 4)	Fisher, Student, Wilcoxon, generalized estimation equation, logistic, Spearman	no
[44]	multicentric, open-label, phase 3, 2000–2004, follow-up of 10 years	adult, PC with one of the following criteria: clinical classification T1b-4 (American Joint Committee on Cancer staging system, 5th edition), Gleason grade 2–6 and PSA > 10 but < 100 ng/mL, T1b-4 Gleason grade seven and PSA < 20 ng/mL, or T1b-1c Gleason score 8–10 and PSA < 20 ng/mL	28 weeks (*n* = 737) vs 8 weeks (*n* = 752) of androgen suppression	PC-specific mortality	overall and disease-free survival, time to loco regional progression or distant metastatic progression, time to biochemical failure and to biochemical failure on secondary androgen suppression, adverse events	no	no
[45]	phase 3, 2002–2006, follow-up of 7 years	favorable- to high-risk PC	hypofractionated external-beam radiotiony (*n* = 151) vs conventional fractionation intensity-modulated radiation (*n* = 152)	cumulative incidence of biochemical and/or clinical disease failure	toxicity (modified LENT and RTOG) criteria, PC mortality, overall survival	no	no
[46]	multicentric, double-blind, phase3, 2006–2010, follow-up of 60 months	pathologically confirmed PC with evidence of bone metastases on a bone scan, judged to be unresponsive or refractory to hormone treatment	atrasentan (*n* = 498) vs placebo (*n* = 496)	progression-free survival and overall survival	toxicities	no (results will be reported separately)	no
[47]	multicentric, double-blind, phase 3, 2009–2010, follow-up of 60 months	PSA progression (PCWG2 criteria) or radiographic progression in soft tissue or bone, ongoing androgen deprivation therapy with a serum testosterone level < 50 ng/dL, ECOG grade 0–1, BPI-SF score 0–3	abiraterone acetate (*n* = 546) vs placebo (*n* = 542)	radiographic progression-free survival and overall survival	time to opiate use for cancer-related pain, long-term safety	no	[27]
[48]	multicentric, double-blind, phase 3, 2008–2011, follow-up of 32 months	histologically confirmed, progressive castration-resistant PC, two or more bone metastases detected on skeletal scintigraphy and no known visceral metastases, baseline PSA level ≥ 5 ng/mL, evidence of progressively increasing PSA values, ECOG grade 0–2, life expectancy ≥ 6 months, adequate hematologic, renal, and liver function	radium-223 (n = 614) vs placebo (*n* = 307)	overall survival	time to first symptomatic skeletal event, defined as the use of external beam radiation to relieve bone pain, or occurrence of a new symptomatic pathological fracture, occurrence of spinal cord compression, occurrence of tumor-related orthopedic surgical intervention	no	[37, 42]
[1]	multicentric, open-label, phase 3, 1994–2013, follow-up of 13 years	aged 50–74 years from population registries and randomly assigned by computer generated random numbers	PSA screening (*n* = 7408) vs no intervention (*n* = 6107)	PC-specific mortality	no	no	no
[49]	multicentric, double-blind, phase 3, 2006–2008, follow-up of 42 months	adult, histologically confirmed, non-metastatic castration-resistant PC at high risk for developing bone metastasis, as characterized by PSA ≥ 8.0 ng/mL within 3 months before random assignment, PSA doubling time ≤ 10 months at baseline, or both	denosumab (n = 716) vs placebo (*n* = 716)	time to first occurrence of bone metastasis or death resulting from any cause	time to first bone metastasis excluding deaths, overall survival	no	no
[50]	phase 3, blind, 2004–2012, follow-up of 36 months	adult, histologically confirmed PC, at least one bone metastasis by radiographic imaging, with ECOG grade 0–2, creatinine clearance (Cockroft-Gault) > 30 mL/min	zoledronic acid (*n* = 323) vs placebo (*n* = 322)	time to first skeletal-related event	overall survival, progression-free survival, safety	no	no
[51]	multicentric, double-blind, phase 3, 2007–2010, follow-up of 54 months	metastatic castrate-resistant PC, adequate organ function, no prior chemotherapy	aflibercept (*n* = 612) vs placebo (n = 612)	overall survival	PSA response, time to skeletal-related event, progression-free survival	no (results will be reported separately)	no
[52]	multicentric, open-label, phase 2, 2009–2011, follow-up of 24 weeks	intermediate- and high-risk patients had histologically confirmed localized PC (> three positive biopsies) and one of the following criteria: PSA > 10 ng/mL, PSA velocity > 2 ng/mL per year, Gleason score > 7	24-week (n = 30) vs 12-week (*n* = 28) abiraterone acetate	12-week prostate tissue testosterone and dihydrotestosterone levels	12- and 24-week intra prostatic hormones, serum hormones, monthly serum PSA, and prostate pathologic assessment	no	no
[53]	multicentric, double-blind, phase 3, 2007–2010, follow-up of 12 weeks	moderate to severe hot flashes ≥ four per day, life expectancy > 9 months, no history of hepatic dysfunction, no allergies to soy or dairy, no uncontrolled hypertension, no history of seizures, no history of intolerance to venlafaxine	venlafaxine + milk powder or placebo + soy powder or venlafaxine + soy powder vs placebo + milk powder	number of hot flashes times severity	12-week QoL level (FACT-P), adverse events	ANalysis Of VAriances	no

### Collected endpoints

Among the 30 papers, only 8 [26–28, 33, 35, 42, 43, 53] were partially based on the collection of Patient Reported Outcomes (PRO). Their median follow-up was 38 months (range from 12 to 52 months) versus 54 months (range from 3 months to 18 years) in the 22 remaining papers. Among the 8 retained papers, six [27, 28, 33, 35, 42, 53] compared the treatments consequences on the patient HRQoL collecting the Functional Assessment of Cancer Therapy-Prostate (FACT-P) questionnaire [55, 56]. The FACT–P is an internationally validated questionnaire specifically designed to assess the HRQoL of men with PC. It is derived from the FACT-General (FACT-G) questionnaire with an additional subscale of 12 items specific to PC (the Prostate Cancer Subscale, PCS). The FACT-G is a 27 items self-report questionnaire measuring general HRQOL in cancer patients (regardless of the tumor type). High FACT-P total score indicates better HRQoL. Note that some indexes are also derived from the FACT–P: the Trial Outcome Index (TOI) based on the physical and functional well-being subscales of the FACT–G and the PCS, and the FACT Advanced Prostate Symptom Index (FAPSI) including eight items from the FACT–P. The two remaining papers compared the interventions in terms of specific PRO: Araujo et al. [26] assessed the patients’ pain with the Short Form of the Brief Pain Inventory (BPI-SF) [57, 58], while Pisansky et al. [43] focused on sexual disorders with the International Index of Erectile Function [59], the Sexual Adjustment Questionnaire [60], and the Locke Marital Adjustment Test [61]. Among the six papers using the FACT-P questionnaire, two papers also employed the BPI-SF questionnaire [27, 35]. Note that only the study proposed by Basch et al. [27] presented a PRO measure (the pain intensity) as primary endpoint. Nevertheless, this paper referred to the same randomized clinical trial initially reported by Ryan et al. [47], which was designed (in particular the sample size determination) by using co-primary endpoints: the radiographic progression-free survival and the overall survival. Therefore, among the 27 trials included in the review, none was specifically designed to analyze the consequences of interventions in terms of HRQoL as a primary endpoint.

### Statistical analyzes used to compare consequences in terms of HRQoL

Among the eight papers including some results related to PRO [26–28, 33, 35, 42, 43, 53], two main strategies were adopted: i) the analysis of the time to HRQoL change, defined as a relative change from baseline higher than a given percentage, or ii) the absolute difference between the HRQoL means at baseline and at a given post-baseline time.

More precisely, the time to HRQoL change was explored in four papers [27, 28, 33, 35]. The statistical analyses were based on the Kaplan-Meier estimator associated with the Log-Rank test or the Cox model. The definitions considered for the time to HRQoL change were heterogeneous:

- In the study by Basch et al. [27], the authors studied the time from baseline to: a 10-point decrease of the FACT-P total score, or a 9-point decrease of the FACT-G score, or a 9-point decrease of the TOI.
- In the study by Beer et al. [28], the authors studied the time from baseline to a 9-point decrease of the FACT-P total score.
- In the study by Fizazi et al. [33], the authors studied two different endpoints: i) the time to deterioration of symptoms in the FAPSI, and ii) the time to deterioration of HRQoL in the FACT-P total score. In the two cases, there was no precision on the used threshold.
- In the study by Fizazi et al. [35], the authors studied the time from baseline to a 10-point decrease of the FACT-P total score or death from any cause, whichever occurred first. Note that the authors compared additional HRQoL endpoints, but without taking into account the time-dependent characteristic of the HRQoL: the percentage of patients with at least a 10-point improvement in the FACT-P total score at any post-baseline assessment and the percentages of patients with at least a 3-point improvement in the five FACT-P subscales (physical wellbeing, social or family wellbeing, emotional wellbeing, functional wellbeing, and PCS). The six percentages were compared by using the stratified Cochran-Mantel-Haenszel test.

In the two remaining studies using the FACT-P, Parker et al. [42] compared the mean change in the FACT-P total score from baseline to week 16 (Student t-test), while Vitolins et al. [53] compared the 12-week HRQoL level by considering six different endpoints (ANalysis Of Variance): the FACT-P total score, the FACT-G score, the social wellbeing, the physical wellbeing, the emotional wellbeing, the functional wellbeing and the PCS.

Interestingly, one can notice that the 8 papers partially based on the PRO collection [26–28, 33, 35, 42, 43, 53] were differentially distributed according to the curative/palliative treatments. Among the 12 papers related to curative treatments, only 1 paper (8.3%) collected PRO [43]. In contrast, among the 18 papers related to palliative treatments, 7 papers (38.9%) collected PRO [26–28, 33, 35, 42, 53].

### Merging the survival and the HRQoL dimensions

All papers analyzed these two dimensions separately, except for two papers [35, 36]. In the study by Fizazi et al. [35], the time to the first event between the HRQoL decrease and the patient death was studied. Heijnsdijk et al. [36] were interested in Quality-Adjusted Life-Years (QALYs) for merging the information about survival and HRQoL in order to perform a cost-effectiveness analysis of PC screening. Nevertheless, in their study, the HRQoL was not individually collected: assumptions were made regarding other data published in the literature.

## Discussion

In the treatment of PC, the most effective intervention in terms of survival may not necessarily be the best one from the patient’s perspective if survival gain involves serious HRQoL deterioration due to treatments side-effects on sexual, urinary and bowel functions. Thus, in randomized clinical trials, it appears important to describe trade-offs between survival and HRQoL. Following this line, the Food and Drug Administration (FDA) has published a guidance document promoting the inclusion of patient-reported outcomes measures in drug development [62]. Moreover, several steps have been identified and proposed for a more patient-centered approach to drug development [63, 64], including patient-centered outcome research which aims to allow the voices of patients to be heard in assessing the value of health care options. In order to evaluate what is currently done in PC clinical research, we performed a mini-review focusing on randomized clinical trials published between 2013 and 2015 in medical journals with a high impact factor.

Among the 30 selected studies, only two papers attempted to merge the patient survival and HRQoL in a single endpoint. The first one, proposed by Fizazi et al. [35], compared the time to the first event between the patient death and the HRQoL deterioration. However, assuming death and HRQoL deterioration are equally important raises questions. The second one, proposed by Heijnsdijk et al. [36], computed QALYs to conduct a cost-effectiveness analysis of PC screening. Although QALYs have been primarily designed for economic evaluation purposes they could also prove useful for clinical decision making [65, 66]. In the late 1990’s the concept of Q-TWIST (Quality-adjusted Time WIthout Symptoms of disease and Toxicity of treatment), which is nearly identical to that of QALYs, has been used by physicians to present the results of PC clinical trials [67, 68]. Broadly speaking, QALYs are computed by assigning to the health states a synthetic HRQoL score, called “utility score”, ranging from zero (death) to one (perfect health) so that each year of life is weighted by the corresponding utility score given the patient’s health state. More precisely, 1 QALY represents 1 year alive in perfect health. For instance, a patient living 10 years with a utility at 0.8 will a have 8 QALYs (10*0.8). This value would be lower for a patient living for 12 years but with an utility at 0.6, the number of QALYs would then be 7.2 (12*0.6) due to a more efficient intervention but with important side effects for example. But the main limitation of the study proposed by Heijnsdijk et al. [36] is that the utility scores used to calculate QALYs were not individually collected during the trial, but they were retrieved from literature.

Among the 30 selected papers, only six papers proposed HRQoL collection but as a secondary endpoint with a short-term follow-up. Two additional papers compared the interventions in terms of specific PRO. This low proportion of PRO-based papers (8/30), is even more dramatic for curative treatments (1/12) compared to palliative treatments (7/18). The analyses of HRQoL were always performed separately from those related to patient survival. This way of presenting results did not allow an interpretation of the potential trade-offs between quantity and quality of life. The shortness of the follow-up in these studies also represents an important limit for balancing between the long-term quantity and quality of life. Moreover, even if six papers used the FACT-P questionnaire, the statistical analyses were highly heterogeneous. For instance, among the four papers in which the time from baseline to HRQoL change was described, the definitions of the HRQoL change were different, and the interval censoring and the informative censoring due to patient death were not taken into account in the analyses. As previously emphasized by Efficace et al. [69], who described that only one-fifth of randomized clinical trials in PC reported adequately PRO data to draw meaningful conclusions, our results indicated that methodological improvements related to HRQoL analyses are essential for a better interpretation by physicians. For instance, Martin et al. [70] have recently provided useful guidelines for better standardizing patient-centered outcomes.

As a matter of fact, specific methodological issues related to PRO analysis do not seem to be considered nor discussed in most of the six PRO-based studies of our review, such as missing data management or choosing a threshold for minimal important change in HRQoL level. Indeed, information on missing data description and analysis is often lacking, which is unfortunate. Such data are likely to be missing not at random, which might lead to biased estimates of treatment effect. Moreover, the choice of thresholds for time to HRQoL change, is either unjustified or refers to the concept of Minimal Clinically Important Difference (MCID) proposed by Cella et al. [55] The latter constituted an important step but it has nevertheless to be outlined that a sample-dependent statistically-based approach was used, which did not rely on the patient’s perspective.

In this mini-review, we voluntary restricted our study to trials published between 2013 and 2015 in medical journals with a high impact factor. This limits the generalizability of the findings. Firstly, we did not include the year 2016, while several important studies have been published. For instance, the ProtecT clinical trial aimed to compare active monitoring, radical prostatectomy, and external-beam radiotherapy for the treatment of clinically localized PC [71, 72]. The authors described separately, in two different papers, the clinical endpoints [71] and the patient-reported endpoints [72]. Again, this illustrates the need of developing future clinical trials that better consider the balance between quantity and quality of life in a single endpoint, such as QALYs. Secondly, many important studies are not published in these journals with high-impact. The researchers who publish in high-impact journals have distinct profiles compared with the researchers who publish in low-impact journals [73], and the cancer trials with positive outcomes are more likely to be published in journals with high-impact [74]. Note also that all main urological journals were not included because of an impact factor lower than 15.

However, the limitations do not disqualify the central message of our mini-review. Our aim was not to propose a complete systematic review, but rather to illustrate the paradox between acknowledging that the treatment choice involves trade-offs between quality and quantity of life and the scarcity of studies that take them into account. Among the 30 selected studies with high-impact, no study precisely describes the potential trade-offs between quantity and quality of life. Based on this result, one can reasonably suggest to further consider composite patient-centered outcomes in future clinical trials, especially for those published in journals with high-impact. Future studies should also take into consideration some psychological aspects that may affect HRQoL [75, 76] and the important role of the family [77].

## Conclusion

In conclusion, our mini-review suggests that recent clinical trials published in journals with high-impact are not designed to precisely describe the potential trade-offs between the quantity and the quality of life. It is now time to avoid designing trials that mainly, or even only, consider clinical efficacy. Composite patient-centered outcomes merging the quantity with the quality of life are needed to propose the most appropriate treatment on behalf of patients’ best interest. We recommend the use of indicators such as QALYs as principal endpoint in future clinical trials.

## Additional file

Additional file 1: The research equation used in PubMed. (DOCX 16 kb)

Additional file 2: The Preferred Reporting Items for Systematic review and Meta-Analysis Protocols (PRISMA-P) checklist. (DOCX 16 kb)

## Abbreviations

BPI-SF: Short Form of the Brief Pain Inventory
FACT-G: Functional Assessment of Cancer Therapy – General (any tumor type)
FACT-P: Functional assessment of cancer therapy for prostate cancer
FAPSI: FACT Advanced Prostate Symptom Index
HRQoL: Health-Related Quality of Life
MCID: Minimal clinically important difference
Mesh: Medical subject headings
PC: Prostate cancer
PCS: Prostate cancer subscale
TOI: Trial Outcome Index
