# Supplementary material for: A mini-review of quality of life as an outcome in prostate cancer trials: patient-centered approaches are needed to propose appropriate treatments on behalf of patients
Source: Health Qual Life Outcomes. 2018 Mar 5;16:40. doi: 10.1186/s12955-018-0870-6 (PMC5836440; doi:10.1186/s12955-018-0870-6)
Supplement: Supplementary file 1 — The research equation used in PubMed. (DOCX 16 kb) [file 12955_2018_870_MOESM1_ESM.docx]

**Additional file 1**

**Request used in PubMed**

(((("2013/05/01"[Date - Publication] : "2015/05/01"[Date - Publication])) AND ((((((((("BMJ (Clinical research ed.)"[Journal]) OR "JAMA"[Journal]) OR "Lancet"[Journal]) OR "The Lancet. Oncology"[Journal]) OR "The New England journal of medicine"[Journal]) OR "CA: a cancer journal for clinicians"[Journal]) OR "Cancer discovery"[Journal]) OR "Journal of clinical oncology : official journal of the American Society of Clinical Oncology"[Journal]) OR "Journal of the National Cancer Institute"[Journal])) AND prostatic neoplasms[MeSH Terms]) AND "randomized controlled trial"[Publication Type]

**PRISMA-P checklist**

**Table S1.** PRISMA-P (Preferred Reporting Items for Systematic review and Meta-Analysis Protocols) 2015 checklist: recommended items to address in a systematic review protocol. Sections “ADMINISTRATIVE INFORMATION” and “INTRODUCTION”.

| Section and topic | Item No | Checklist item | Precision |
| --- | --- | --- | --- |
| ADMINISTRATIVE INFORMATION | | |  |
| Title: |  |  |  |
| Identification | 1a | Identify the report as a protocol of a systematic review | We qualified our study as a mini-review, i.e. a systematic but focused review |
| Update | 1b | If the protocol is for an update of a previous systematic review, identify as such | This is not an update |
| Registration | 2 | If registered, provide the name of the registry (such as PROSPERO) and registration number |  |
| Authors: |  |  |  |
| Contact | 3a | Provide name, institutional affiliation, e-mail address of all protocol authors; provide physical mailing address of corresponding author | We provided these details in the first page |
| Contributions | 3b | Describe contributions of protocol authors and identify the guarantor of the review | We provided these details in the subsection entitled “Authors' contributions” |
| Amendments | 4 | If the protocol represents an amendment of a previously completed or published protocol, identify as such and list changes; otherwise, state plan for documenting important protocol amendments | Not applicable |
| Support: |  |  |  |
| Sources | 5a | Indicate sources of financial or other support for the review | We provided the financial support in the subsection entitled “Funding” |
| Sponsor | 5b | Provide name for the review funder and/or sponsor | We did not have additional sponsors |
| Role of sponsor or funder | 5c | Describe roles of funder(s), sponsor(s), and/or institution(s), if any, in developing the protocol | The funder has no role in our research. |
| INTRODUCTION | | |  |
| Rationale | 6 | Describe the rationale for the review in the context of what is already known | The rational is described in the introduction. |
| Objectives | 7 | Provide an explicit statement of the question(s) the review will address with reference to participants, interventions, comparators, and outcomes (PICO) | We define the objective of this review in the introduction |
