# Supplementary material for: A mini-review of quality of life as an outcome in prostate cancer trials: patient-centered approaches are needed to propose appropriate treatments on behalf of patients
Source: Health Qual Life Outcomes. 2018 Mar 5;16:40. doi: 10.1186/s12955-018-0870-6 (PMC5836440; doi:10.1186/s12955-018-0870-6)
Supplement: Supplementary file 2 — The Preferred Reporting Items for Systematic review and Meta-Analysis Protocols (PRISMA-P) checklist. (DOCX 16 kb) [file 12955_2018_870_MOESM2_ESM.docx]

**Additional file 2**

**Table S2.** PRISMA-P (Preferred Reporting Items for Systematic review and Meta-Analysis Protocols) 2015 checklist: recommended items to address in a systematic review protocol. Section “METHODS”.

| Section and topic | Item No | Checklist item | Precision |
| --- | --- | --- | --- |
| METHODS | | |  |
| Eligibility criteria | 8 | Specify the study characteristics and report to be used as criteria for eligibility for the review | This information is presented in the subsection entitled “Literature Search Strategy” |
| Information sources | 9 | Describe all intended information sources with planned dates of coverage | We indicated in the subsection entitled “Literature Search Strategy” that PubMed was the only source |
| Search strategy | 10 | Present draft of search strategy to be used for at least one electronic database, including planned limits, such that it could be repeated | The request used in PubMed was detailed in Appendix |
| Study records: |  |  |  |
| Data management | 11a | Describe the mechanism(s) that will be used to manage records and data throughout the review | We used Zotero to manage the records & Excel to collect the data |
| Selection process | 11b | State the process that will be used for selecting studies (such as two independent reviewers) through each phase of the review | The selection process is detailed in the subsection entitled “Data Extraction” |
| Data collection process | 11c | Describe planned method of extracting data from reports (such as piloting forms, done independently, in duplicate), any processes for obtaining and confirming data from investigators | As explained in the subsection entitled “Data Extraction”, the data extraction was performed independently by two reviewers |
| Data items | 12 | List and define all variables for which data will be sought (such as PICO items, funding sources), any pre-planned data assumptions and simplifications | The list of extracted variables corresponds to the columns of the Table 1 |
| Outcomes and prioritization | 13 | List and define all outcomes for which data will be sought, including prioritization of main and additional outcomes, with rationale | We collected all the studied outcomes presented in the selected papers |
| Risk of bias in individual studies | 14 | Describe anticipated methods for assessing risk of bias of individual studies, including whether this will be done at the outcome or study level, or both; state how this information will be used in data synthesis | Not applicable (We did not performed quantitative synthesis) |
| Data synthesis | 15a | Describe criteria under which study data will be quantitatively synthesised |  |
|  | 15b | If data are appropriate for quantitative synthesis, describe planned summary measures, methods of handling data, etc. |  |
|  | 15c | Describe any proposed additional analyses |  |
|  | 15d | If quantitative synthesis is not appropriate, describe the type of summary planned |  |
| Meta-bias(es) | 16 | Specify any planned assessment of meta-bias(es) | We have described the possible biases of our review in the discussion |
| Confidence in cumulative evidence | 17 | Describe how the strength of the body of evidence will be assessed | As discussed at the end of the paper, our conclusions are restricted to trials published between 2013 and 2015 in medical journals with a high impact factor |
